# Airflow and Air Velocity Measurements While Playing Wind Instruments, with Respect to Risk Assessment of a SARS-CoV-2 Infection

**DOI:** 10.3390/ijerph18105413

**Published:** 2021-05-19

**Authors:** Claudia Spahn, Anna Maria Hipp, Bernd Schubert, Marcus Rudolf Axt, Markus Stratmann, Christian Schmölder, Bernhard Richter

**Affiliations:** 1 Freiburg Institute of Musicians’ Medicine, University Medical Center Freiburg, University of Music Freiburg, Medical Faculty of the Albert-Ludwigs-University Freiburg, 79110 Freiburg i.Br., Germany; claudia.spahn@uniklinik-freiburg.de (C.S.); bernhard.richter@uniklinik-freiburg.de (B.R.); 2 Tintschl BioEnergie und Strömungstechnik AG, Tintschl Unternehmensgruppe, 91058 Erlangen, Germany; bernd.schubert@tintschl.de; 3 Bamberg Symphony, Bavarian State Philharmonic Orchestra, 96047 Bamberg, Germany; marcus.axt@bamberger-symphoniker.de (M.R.A.); markus.stratmann@bamberger-symphoniker.de (M.S.); christian@schmoelder.de (C.S.)

**Keywords:** SARS-CoV-2 pandemic, performance studies, dispersion of airborne transmission

## Abstract

Due to airborne transmission of the coronavirus, the question arose as to how high the risk of spreading infectious particles can be while playing a wind instrument. To examine this question and to help clarify the possible risk, we analyzed 14 wind instruments, first qualitatively by making airflows visible while playing, and second quantitatively by measuring air velocity at three distances (1, 1.5, 2 m) in the direction of the instruments’ bells. Measurements took place with wind instrumentalists of the Bamberg Symphony in their concert hall. Our findings highlight that while playing, no airflows escaping from any of the wind instruments—from the bell with brass instruments or from the mouthpiece, keyholes or bell with woodwinds—were measurable beyond a distance of 1.5 m, regardless of volume, pitch or what was played. With that, air velocity while playing corresponded to the usual value of 1 m/s in hall-like rooms. For air-jet woodwinds, alto flute and piccolo, significant air movements were seen close to the mouthpiece, which escaped directly into the room.

## 1. Introduction

The coronavirus pandemic has had and continues to have a grave impact on music making, especially concerning the playing of wind instruments and singing. Airborne transmission plays an important role in the spread of the SARS-CoV-2 virus [1]. Thus, forms of musical sound generation that involve breathing are suspected of being risky. In this regard, it is important to learn more about airflow and air velocity produced by playing wind instruments and singing that could contain infectious droplets or aerosols and spread them in indoor situations. It is therefore necessary to first understand the basics of air dispersion in individual musicians in order to adapt risk assessments during playing.

Research to date has looked into air dispersion while playing wind instruments or singing using different methods of measuring.

In a recent publication conducted at the Bauhaus-Universität Weimar [2], the spread of breathing air while playing wind instruments and singing was observed using Schlieren imaging with a Schlieren mirror and the Background Oriented Schlieren method (BOS) as a way to make respiratory air visible by use of density gradients [3]. Two professional singers (baritone and soprano) and eleven wind instruments (woodwinds: oboe, bassoon, Bb clarinet, bass clarinet, flute, piccolo and alto flute; and brass: Bb trumpet, tenor trombone, French horn and F tuba) of the professional orchestra Thüringen Philharmonic Gotha, Eisenach, were positioned in front of the Schlieren mirror while playing or singing. The findings show that the spreading range as well as the angle at which the air escapes the mouth or outlet vary strongly among instruments and players, depending on the structure of the instrument, the structure of the mouthpiece, the way an instrument is blown and individual blowing or breathing capacities. In general, their measurements with wind instruments reached a maximal distance of 1.12 m, measured by side-air movements of the piccolo.

The authors also discovered that special barrier caps—used with brass instruments—do have a significant impact on the spread of air, which can strongly reduce the dispersion while hardly interfering with the sound of the instrument. Furthermore, they found out that the escaping air ascends due to natural convection or mixes with the surrounding room air [2].

A study by the Ludwig-Maximilians-Universität München and the University of Erlangen [4], pre-published in July 2020, investigated different forms of speaking and singing with 10 professional singers of the Bavarian Radio Choir. They made respiratory flows visible by exhaling smoke of e-cigarettes (containing no nicotine). Tests were conducted in a shaded room, documenting the exhaled aerosol clouds with the help of a high-speed camera and laser light. Comparing different settings (singing text, speaking text and singing without text, once with soft and once with loud phonation), they learned that singing text and speaking text reach comparable mean distances of dispersion up to 0.85 m, and singing without text reached the lowest value of airflow at 0.63 m. Even though the mean measurements of air dispersion stayed within a range of 1 m, some singers reached airflow distances of up to 1.4 m. These measurements were then compared to coughing, which turns out to reach farther distances, with a mean of 1.3 m and a maximum of 1.9 m. On the basis of their study, they suggested a distance regulation of at least 2 m to the front.

Their second study on wind instruments using the same test setting was also published online [5]. They pointed out that respiratory clouds to the front reach farther than to the side, with instrument-specific outcomes. For an alto flute they recommend 3 m spaces to the front and 2 m to the side; for all other wind instruments they recommend 2 m to the front and 1.5 m to the side.

Another study conducted by the University of the German Armed Forces in Munich [6], which was pre-published in May 2020, analyzed larger droplets when singing and speaking, as well as flow-related small droplets when singing and playing wind instruments. The study was conducted with a professional singer, two amateur choir singers, five professional musicians (clarinet, flute, oboe, bassoon and trumpet) and an amateur brass player (trumpet, trombone and euphonium). The motion of droplets and air leaving either the mouth or the outlet was observed during exhalation, which was then illuminated with a laser and recorded with a digital camera, producing a series of images that were subsequently quantitatively analyzed. The analysis pointed out that while singing airflow was no longer detectable at a distance of 0.5 m, regardless of volume, pitch or whether the singer was a professional or an amateur. The analysis of brass instruments showed strong air movements in front of the instrument, which did not reach farther than 0.5 m. Woodwinds produced comparably more airflow, reaching a distance of around 1 m. Concluding, they recommend a minimal radial distance of 1.5 m between singers and wind instrumentalists.

In addition, there was study by Parker and Crookston [7] (pre-published in July 2020) which measured aerosols while playing brass instruments and singing. The authors analyzed seven brass instruments (cornet, horn, baritone, euphonium, trombone, Eb tuba and Bb tuba), investigating the effect that playing for a more extended period of time has on the release of particles in comparison to singing, breathing and using a special barrier cap. To investigate the particles released, they were size sorted and counted with a six-channel laser particle counter. It was discovered that breathing produces more respiratory droplets than playing, and the authors stated that the use of a barrier reduces the release of aerosols by 95%. They also found that within a period of time the production of aerosols increases again and fresh air exchange is required.

At the University of Minnesota, a study on aerosol generation by different wind instruments analyzed 15 musicians from the Minnesota Orchestra [8] (playing the trumpet, bass trombone, French horn, tuba, piccolo, bassoon, oboe, clarinet and bass clarinet) while playing their instruments, breathing and speaking. The aerosol concentration and size was measured using an aerodynamic particle sizer. In comparison to the aerosol generation while speaking and breathing, the instruments were thereafter categorized into low, intermediate and high-risk levels. They pointed out that air-jet instruments (piccolo and flute) produced half the aerosols at the outlet and the other half near the embouchure. The bassoon also produces aerosols at the keyholes and the bell. For woodwinds in general, the mouthpiece and tube structure play significant roles in the generation of aerosols. They found that for brass instruments the total length of the tube correlates with the concentration of aerosols (trumpet > bass trombone > French horn > tuba).

Furthermore, Mürbe et al. [9] pre-published a study on the increase of aerosols during professional singing. Testing eight professional singers (two female sopranos, two female altos, two male tenors and two male baritones) of the RIAS chamber choir Berlin, they measured particle emission rates with the help of a laser particle counter during breathing, reading, singing and holding a long tone. They confirmed their assumption that singing produces higher emission rates than speaking, with mean measurements of 4.71–84.76 P/s during speaking and 753.4–6093.14 P/s during singing. Women also produced higher particle emission rates than men, leading to the assumption that high voices produce a higher sound pressure level than lower voices.

In consideration of their study, Mürbe et al. [10], Kriegel and Hartmann [11] and Hartmann et al. [12] published various risk assessments on the risk of infection with virus-loaded aerosols while singing indoors during the SARS-CoV-2-pandemic, assuming that different styles of singing—e.g., singing vs. speaking—as well as different intensities of voice can lead to various sizes and densities of droplets and aerosols [9], and that room situations for choir rehearsals have to be taken into account [11,12].

They also found out that children’s voices emit fewer aerosols during singing than adult’s voices [13]. The study tested eight children (four girls and four boys) of semi-professional children’s choirs (Staats-und Domsingknaben Berlin and a girl’s choir of the Berliner Singakademie), who were all 13 years old (except for one girl who was 15 years old). The study was conducted the same as their previous study on professional adult singers as mentioned above. Their mean measurements showed emission rates of 16–267 P/s for speaking, 141–1240 P/s for singing and 683–4332 P/s for shouting.

These up-to-date studies looked into the spreading of air or aerosols while playing wind instruments or singing, focusing on different instruments or singing styles, or using different forms of measuring, and making airflows visible. They give similar results focusing on the visualization of airflows while playing wind instruments and singing. Accordingly, different methods were used—the Schlieren method, to make respiratory airflows visible [2]; for the visualization of airborne transmission of professional singers and wind instruments, e-cigarette smoke [4,5]; for the observation of large and small droplets from wind instruments and singers, illuminating airflows with laser and analyzing picture series [6]; for the release of respiratory aerosols while speaking and playing brass instruments, and for using barrier caps, the generation of aerosols of different wind instruments was measured with an aerodynamic particle sizer [7]; and for the measurement of particle emission rates of professional singers [9] and children [13], a laser particle counter—leading to several risk assessments of singing indoors during the SARS-CoV-2-pandemic [10,11,12].

At the time this study was conducted (May 2020), the distance regulation of 12 m between wind instruments was recommended by official instructions and statutory accident insurance. These circumstances made clear that scientific evidence of air dispersion for musicians was urgently needed. With this background it was tremendous to bring evidence into the field of music making. In this respect, our study aimed to provide funded data on velocity, direction and distance of respiratory air while playing wind instruments.

## 2. Materials and Methods

### 2.1. Sample

Players of brass instruments (trumpet, trombone, horn and tuba) and woodwind players (alto flute, piccolo, oboe, English horn, clarinet, bass clarinet, bassoon and contrabassoon) of the Bamberg Symphony voluntarily took part in the study. Two further professional musicians—tenor saxophone and recorder players who were not part of the classical orchestra—were also included, leading to a total sample of 14 wind instrument players.

Due to ethical reasons, all persons were asked by the administration of the Bamberg Symphony to take part in the measurements. At this time of lockdown, musicians were highly motivated to contribute to research in order to make playing possible again. Consent for the study was given by the Ethics Committee of the Universitätsklinikum Freiburg.

In order to exclude infectious persons, before the measurements all players were questioned if they had typical symptoms of the Covid-19 disease. Additionally, everyone’s temperature was taken with an electric fever thermometer directly before entering the concert hall. No one indicated suspicious symptoms and the measurements of the temperature showed values under the cut-off of 37.5 °C in all persons.

### 2.2. Design and Procedure

At the beginning of May 2020 measurements were conducted that were initiated by the Bamberg Symphony Orchestra during the first lockdown of the Covid-19 pandemic in Germany. All players included were examined while playing their instruments.

**Figure A1:**
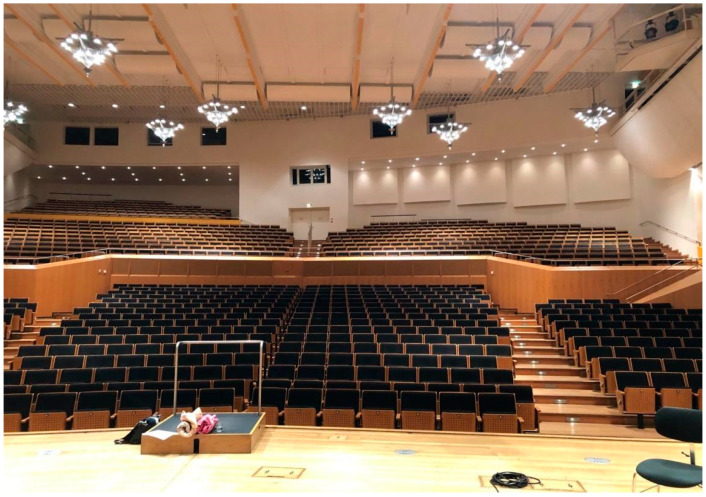
Test location, concert hall of the Bamberg Symphony.

**Figure A2:**
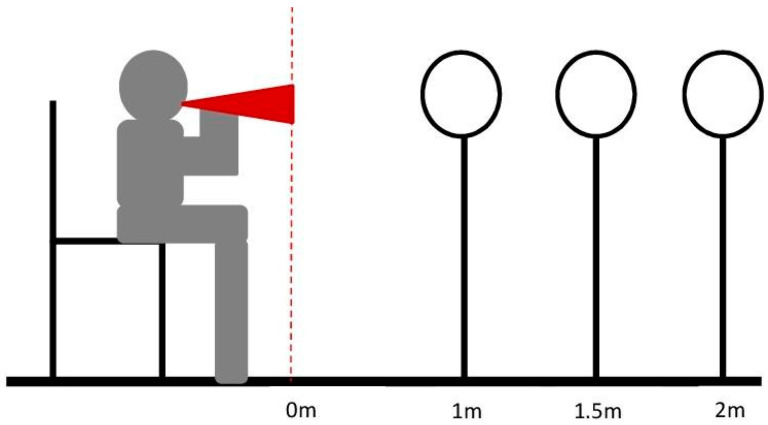
Distance measurements with three sensors in the direction of the instrument’s bell.

**Figure A3:**
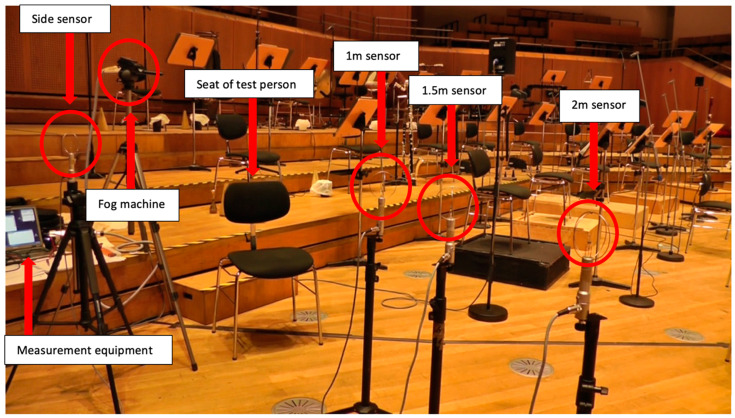
Test set-up on stage of the Bamberg Symphony Hall.

The measurements took place in a typical environment for classical musicians using the stage of the Bamberg Symphony (see Appendix A, Figure A1). The aim of the measurements was to first make respiratory air visible and then to measure air velocities at and coming from the instruments’ bell or other outlets (e.g., keyholes or side-air). We wanted to find out where air velocities can be measured, at what values and to what extent. Therefore, air velocity sensors were put at the bell of the instrument at distances of 1, 1.5 and 2 m (see Appendix A, Figure A2 and Figure A3). For the qualitative analysis, the measurements were also filmed on a digital camera. For all wind instruments except the recorder, qualitative and quantitative measurements could be analyzed. Due to technical disruptive factors for the recorder, only qualitative observations are available.

All wind instrumentalists played scales, excerpts of music pieces, and long tones with different pitches and dynamics (pp–ff), as well as different articulations (e.g., staccato, tenuto, or legato). Being particular to each instrument, the measurement focused specifically on the escape of air through tone holes and outlets. Since warming up is different for every instrument, and brass players usually include a lot of blowing through the mouthpiece (without using the whole instrument), these situations were analyzed as well.

### 2.3. Visualization of Airflow

The flow visualization by use of artificial mist, as used in this study, is one option for on-site-inspection of airflows. Using the technique of flow visualization with FlowMaker™, swirls have been qualitatively made visible at the outlets of wind instruments [14,15,16].

A harmless artificial fog, named SAFEX (the Günther Schaidt SAFEX^®^-Chemie GmbH, Tangstedt, Germany) [17], which consists of water droplets, was used in this study. The use of this fog is especially designed for this kind of scientific use and not comparable with, e.g., proper stage fog. It has, therefore, special components, following a German norm, and loses its measurability after 5–10 cm. The fog is blown out of the fog machine, but its start-push velocity does not reach further than 5–10 cm/s. The cloud of artificial fog that can be seen in front of the test person was merely colored surrounding air, and did not have measurements of its own.

The fog machine was placed next to the musician and always pointed at the instrument’s bell, which was usually positioned 40–50 cm away. Furthermore, the fog machine was knowingly positioned parallel to the measurement sensors in the direction of the instrument’s outlet, and could therefore not blow directly into the velocity measurement sensor.

**Figure A4:**
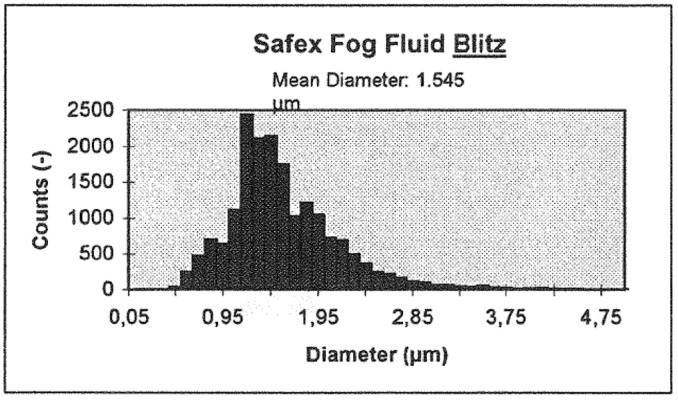
Size distribution of droplets (measurements with DANTEC PDA).

The fog droplets had a size smaller than 5 µm (see Appendix A, Figure A4) and can be compared to the dangerous core droplets of the coronavirus [16].

**Figure A5:**
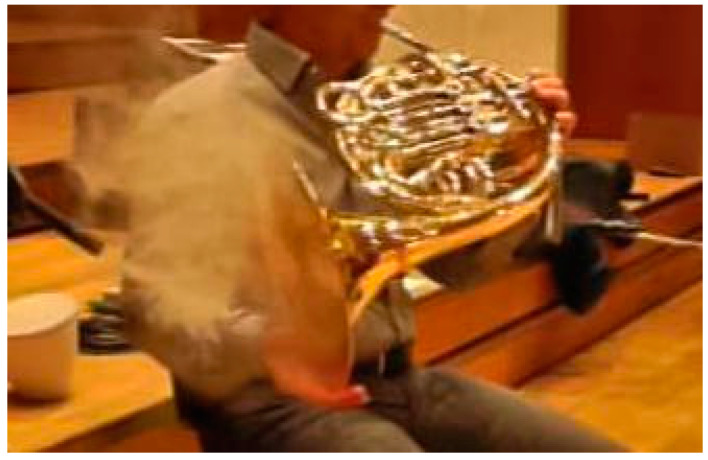
Artificial fog at the bell of the horn.

The artificial fog was transported through a system called “Hydra”, using a flexible tube, to the release spots of the instruments—embouchure area, bell and key openings. Through the application tube installed on a stand, the fog escaped into the free space of the room and created a cloud of fog. It was oriented towards the musicians, who placed the outlet of their instrument directly into the cloud (see Appendix A, Figure A5).

The movement of the fog was filmed with a video camera and qualitatively analyzed afterwards.

### 2.4. Measurement of Air Velocity (Anemometry)

For the measurement of air velocity, omnidirectional (independent of direction) hot film probes of type DISA 54N50 Low Velocity Air Flow Analyzer, manufactured by DANTEC, were used. The probes have a measuring area of 0–1 m/s with an accuracy of 0.2–0.4% full scale. The corresponding electronics (LVFA) give a linear voltage signal of 0–2 Volts, according to its velocity, which is recorded with a 20-bit AD converter in the computer. Before the measurements, the measurement chain (sensor-electronic measurement equipment-signaling cable-converter-computer) was verified in the company-owned wind tunnel against a laser-Doppler anemometer.

With distances of 1, 1.5 and 2 m from the exhaust opening, ball tubes were put on stands and placed in a line. All sensors were at a height of 1 m above ground and were adjusted according to the different instruments and their outlet holes (see Appendix A, Figure A3).

The linear output signal of the controller was mapped with a 20-bit AD converter and recorded on the cable-connected computer every second.

The relation of the measuring signal and the actual velocity was verified according to the measurements of the in-house calibration wind tunnel, in the area of 0.15–0.7 m/s.

Parallel to the measurements of the velocity, video recordings with a manual camera were also conducted.

**Figure A6:**
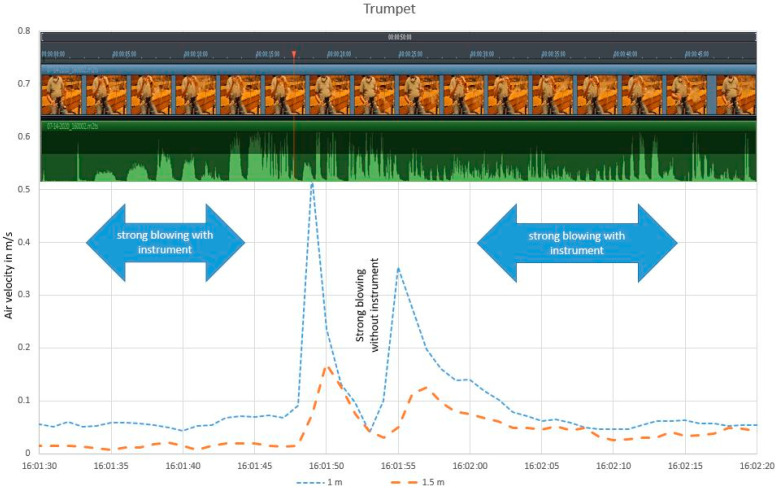
Example measurements (trumpet): blowing, with and without the instrument. Note: This is an example of the measurements (trumpet). Screenshots from the qualitative videos can be seen at the top, followed by the waveform of the audio recording in green. It shows the different measurements of airflows between warming up (when the player blows into the instrument’s mouthpiece) and blowing long tones with the instrument. At 2 m no more airflows were measurable; therefore, the 2 m line is irrelevant and not included in the graphic.

The quantitative measurements were used to support the qualitative observations by focusing the distances of 1, 1.5 and 2 m and measuring air velocities in direction of the instruments’ outlets. The video sequences of the qualitative observations were timed with the measurements of the velocity measurement probes to get the relations of distance, direction and velocity of the emitted air. Hereinafter, the findings are presented by selected measuring charts (e.g., see Appendix A, Figure A6). These charts were used as the basis for the analysis and were compared to data numbers and video sequences in order to understand what air velocities were measured where and while doing what kind of playing or warming up.

### 2.5. Measuring Setup and Location

Among circles of experts on air technology, room air velocities are part of indoor climate discussions. Airflows are measured in m/s and set in relation to each other in different room situations. Thresholds of air movements below 0.1 m/s are understood to be random air circulations, necessary in every habitable room in order to ensure fresh-air supply [18]. They can be described as “background noises.” Hence, only airflows above a threshold of 0.1 m/s can be used systematically, making the threshold of 0.1 m/s a physical boundary for airflow measurements [18].

Furthermore, the indoor climate of habitable rooms is called comfort climate: “A climate of comfort persists when people feel thermally at ease in habitable rooms” [18] (p. 6). Whether a person is feeling comfortable within a room is—amongst other components, such as temperature—dependent on air velocities, since a comfortable climate is free from draught.

Draught is the undesired cooling of the body through air movement. It can be felt from a value of 0.15 m/s, and depends on the size of a room and its ventilation system [18]. The perception of comfortableness of concert halls is therefore closely related to ventilation systems of the hall. So-called “well-like ventilations” coming from the ground are usually used nowadays [19]. In this system, the velocity at which the air exits the ventilation system has to be taken into account, since it is regulating how comfortable the audience feels. For concert halls, exit velocities of 0.2 m/s are recommended (in relation to a room temperature of 20°), to not surpass an air velocity of 0.15 m/s at the height of 1 m (where the audience is sitting) and to stay in the comfort zone of 0.1–0.2 m/s [18]. Hence, for concert halls, which have a room temperature of 20°, room air velocities of 0.1–0.16 m/s are usually estimated.

Looking at the circumstances of the concert hall of the Bamberg Symphony, the ventilation system was analyzed in 2017 to understand draught appearances on stage, giving our study funded data on the room air conditions, including the room temperature of 22.3°. Hence, the draught risk—where people start to feel uncomfortable—was made maximal at 0.15 m/s, and we therefore considered an area of comfortableness of 0.1–0.15 m/s [15].

These numbers underline that measurements under 0.1 m/s cannot be recognized by people. Simultaneously, they point out that 0.15 m/s is already felt as a draught, leading to a range of room comfortableness between 0.1 and 0.15 m/s. 

Looking at wind instruments, air velocities under a threshold of 0.1 m/s are not considered, whereas values of 0.15 m/s and above can be understood as remarkable.

**Figure A7:**
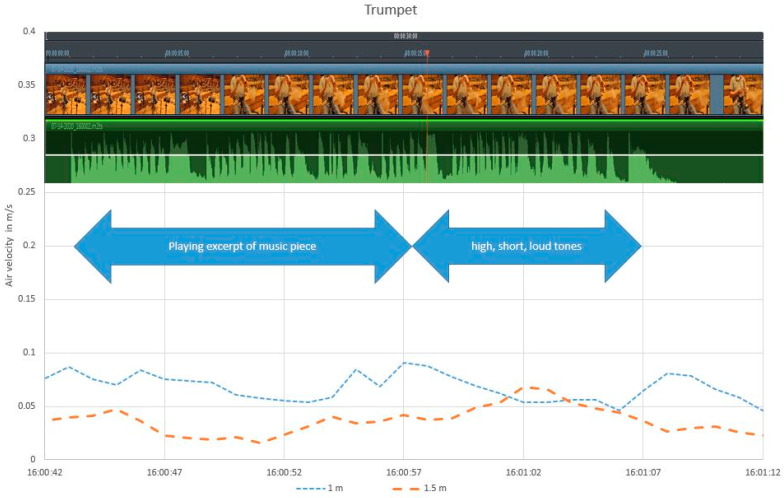
Example measurements 2 (trumpet): playing and pitches. Note: This is an example of the measurements (trumpet). At the top, screenshots from the qualitative videos can be seen and the acoustics are shown. The graphic shows that there are no differences in the measurements of airflows between playing excerpts of a music piece or short high pitches. At 2 m no more airflows were measurable; therefore, the 2 m line is irrelevant and not included in the graphic.

As a basis for the analysis, graphs for every instrument were produced, indicating the findings of an instrument. These charts compare the airflow visualizations of the video sequences to the measured numbers of airflows within the three distances (1, 1.5, 2 m) in direction of the instruments´ outlets (see Appendix A, Figure A6 and Figure A7).

Making use of the descriptive analysis, the measurements of every instrument were also put into a table, comparing air velocities of different instruments at the three different distances. This overview shows the maximum values of every instrument at every distance—which were compared to the numbers of room comfortableness. Air velocities produced by a wind instrument that are under a value of 0.1 m/s do not have an impact on the compartment air and “disappear” amongst “background noises,” meaning velocities. On the other hand, air velocities with a value over 0.3 m/s are comparable to strong draughts or coughing and therefore have a strong impact on dispersion of air, and on the dispersion of core droplets, such as SARS-CoV-2 virus droplets.

## 3. Results

### 3.1. Qualitative Flow Visualization by Use of Artificial Fog

**Figure A8:**
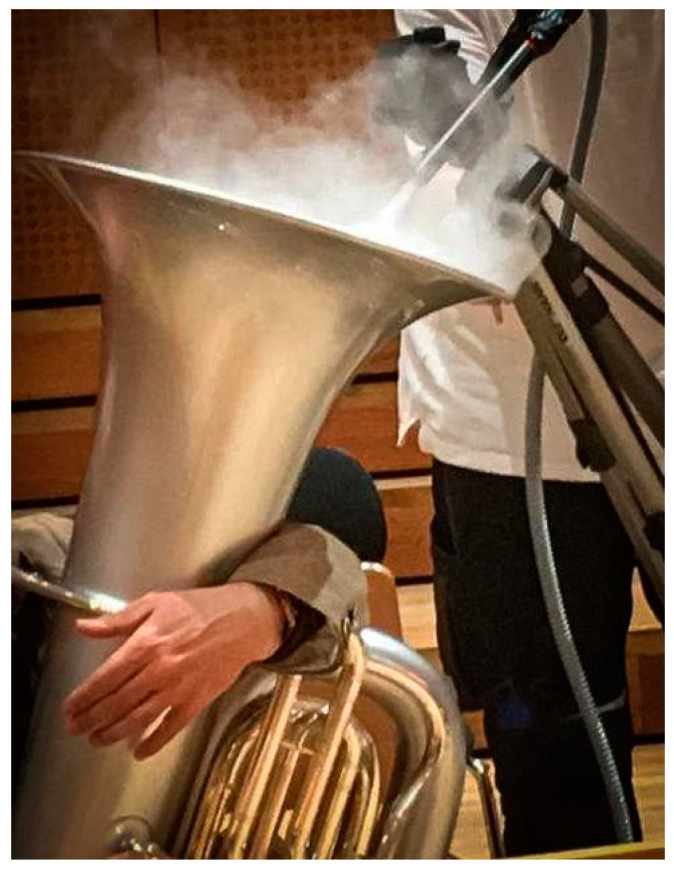
Artificial fog at the bell of the tuba.

The qualitative observations served the analysis of airflows during playing (see Appendix A, Figure A8) and gave initial insights into the movement of airflows from the different outlets of the instruments. Aside from that, these observations were significant as to the positions of the sensors for the air velocity measurements, showing precisely at what points on the instrument airflows escaped. The sensor of the tuba, for example, was positioned above the instrument, since its outlet directs upwards, whereas the outlet of the oboe points to the floor and the output of the horn points backwards. The main outlet for the observed brass instruments (trumpet, trombone, horn and tuba) was the bell, whereas for woodwinds, keyholes as well as airflows close to the mouthpiece have to be considered.

#### 3.1.1. Brass

Next to airflows that escaped at the bell of the brass instruments, further air movements were seen while deflating the instrument (blowing out condensed water), which concerned mainly the trumpet and trombone. This procedure is part of warming-up and generates visible airflows that can only be seen within a distance of 1 m. Comparing these air movements to directed blowing (in direction of the sensors), blowing without the instrument showed stronger and faster air movements in the artificial fog. While the trumpet and trombone players were playing an excerpt of a music piece, only very small air movements were made visible, which mixed with the surrounding room air velocity quickly. The tuba and horn made no visible air movements while playing.

#### 3.1.2. Air-Jet Woodwinds

For the piccolo and alto flute, side airflows could be seen, escaping the mouth close to the mouthpiece, directly reaching to the ground and staying visibly close to the player’s body. Aside from these significant observations, no further airflows could be seen at the bells of these two air-jet instruments.

**Figure A9:**
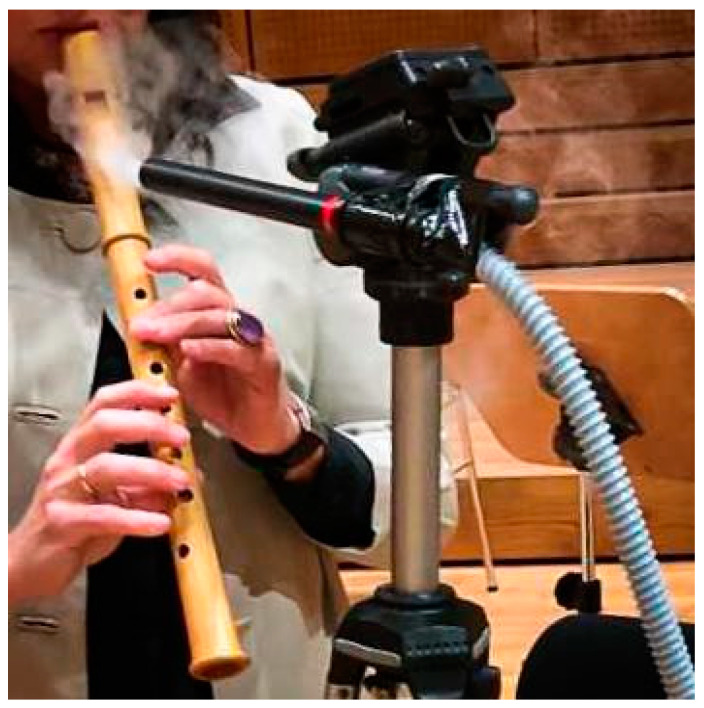
Artificial fog at the labium of the recorder.

With the recorder (early baroque in g, and soprano) small air movements were visible at the labium of the instrument at a distance of 20 cm at maximum (see Appendix A, Figure A9). Further airflows were not visible at the bell of the recorder, leading to the presumption that the labium can be understood as the instrument’s main outlet.

#### 3.1.3. Reed Woodwinds

Further reed woodwind instruments that were measured (single reeds: clarinet, bass clarinet, saxophone and double reeds: bassoon, contra bassoon, oboe and English horn) mainly showed airflows escaping the instrument’s bell, and some at the keyholes. Even though these instruments showed airflows escaping from the keyholes as well, these air movements showed only minor dispersions in the artificial fog.

While playing the clarinet, for example, small airflows were visible at the bell of the instrument, which were thereafter compared to blowing without the instrument. This comparison showed how small the air movements with the instrument were, in comparison to the fast and strong airflows produced by blowing from the mouth of the clarinetist without the instrument.

### 3.2. Measurement of Air Velocity (Anemometry)

The air velocity measurements with all wind instruments mostly did not surpass a value of 0.1 m/s (see Table 1) while playing an excerpt of a music piece, scales or different pitches and volumes. Even though air movements were qualitatively seen at the bells of some wind instruments, these qualitative observations did not reach measurements of more than 0.1 m/s, which is the value of usual room air velocities in hall-like rooms. Hence, some little air movements were measurable at the 1 m sensor (concerning tuba: 0.13 m/s; oboe: 0.15 m/s; and contrabassoon: 0.11 m/s—at the 1 m sensor), not surpassing measurements of 0.1 m/s at the 1.5 or 2 m sensors.

#### 3.2.1. Brass Instruments

Playing a trumpet, a trombone, a horn or a tuba showed only little or no airflows in the artificial fog. However, there was a difference between playing and warming up. Since brass players often use only mouthpieces while warming up, direct airflows are produced, which can reach measurements higher than 0.1 m/s. In comparison, loudness or pitch did not have an impact on the air velocities (see Appendix A, Figure A6 and Figure A7).

##### Trumpet

While playing different scales, high and medium pitches, all measurements of the trumpet stayed under 0.1 m/s at any distance, which corresponds with the usual room air velocity.

However, when the player deflated the instrument, air velocities reached 0.14 m/s at a distance of 1 m from the bell, while at 1.5 and 2 m they stayed under 0.1 m/s. Comparing these numbers to the values of blowing without the instrument, the measurements were much higher, producing air movements of 0.52 m/s to the front at a distance of 1 m. At 1.5 m during blowing without the instrument, they dropped to 0.15 m/s and at a distance of 2 m they stayed under 0.1 m/s.

##### Trombone

Similarly to the measurements of the trumpet, air velocities of the trombone did not reach significant values (see Table 1) while playing scales and different pitches, or even while deflating at any distance.

When the person was only blowing, without the instrument or the mouthpiece, the highest measurements were acquired, reaching values of 0.4 m/s at 1 m, 0.18 m/s at 1.5 m and 0.1 m/s at 2 m in front.

##### Horn

For the horn, no meaningful airflow measurements were made at any of the three distances (see Table 1), regardless of whether they were playing an excerpt from a music piece, different pitches or volumes.

##### Tuba

The tuba did reach the highest measurements of all brass instruments during playing a music piece or warming up with the instrument. While playing scales and while deflating, they did not surpass a value of 0.1 m/s at every distance. However, when the player did an excerpt of a music piece or warmed up the instrument, the measurements reached 0.13 m/s at a distance of 1 m or closer (in the direction of the bell), and stayed again under 0.1 m/s at 1.5 and 2 m.

#### 3.2.2. Woodwind Instruments

For some woodwinds, the measurements indicate that air movements were not only visible at the bell of the instrument, but also at other outlets, such as side-air observations or airflows escaping tone holes, when comparing air-jet woodwinds and reed woodwinds. There were no significant differences in the measurements for single reed and double reed woodwinds.

##### Air-Jet Woodwinds

Since there were qualitative observations of side-air movements for both air-jet woodwinds, another sensor was put up at 0.5 m to the side of the player, measuring side-air velocities.

Alto Flute

Regardless of what the flute player was playing, all measurements to all sides and distances stayed under 0.1 m/s. The additional measurements to the side reached a value of 0.15 m/s at a distance of 0.5 m.

Piccolo

The measurements at the bell of the piccolo were not significant (see Table 1) and therefore mixed with the surrounding room air velocity of the concert hall. Other than the flute, side-air measurements for the piccolo showed a slightly smaller value of 0.13 m/s.

##### Single Reed Woodwinds

The qualitative observations showed air movement at the keyholes of several reed woodwinds, which were considered due to the front sensor at 1 m.

Clarinet

No significant air movements coming from the clarinet (at all distances) were measurable, regardless of whether they were playing scales, different pitches or an excerpt from a music piece.

Bass Clarinet

Air velocity measurements for the bass clarinet stayed under a threshold of 0.1 m/s, with no difference depending on what was played (an excerpt from a music piece, long tones or staccato), at distances of 1, 1.5 and 2 m.

Tenor Saxophone

The airflow measurements of the tenor saxophone did not reach a significant threshold above 0.1 m/s at any distance (see Table 1)—regardless of what the player was playing.

##### Double Reed Woodwinds

Oboe

The measurements of the oboe reached a value of 0.15 m/s at 1 m, 0.12 m/s at 1.5 m and stayed under the usual room air movement of 0.1 m/s at 2 m.

Bassoon

All measurements of the bassoon stayed under a value of 0.1 m/s at all distances, regardless of what was played.

Contrabassoon

All measured data of the contrabassoon were insignificant concerning meaningful airflows, with no differences between long tones or an excerpt from a music piece at any distance from the bell.

English Horn

While playing the English horn, regardless of what was played, all measurements in the direction of the bell stayed under the significant threshold of 0.1 m/s.

### 3.3. Impacts of a Ambient Noises

As a counter phase to the individual measurements, situations with ambient noises were also considered, since they happen frequently during orchestra rehearsals or concerts, e.g., while setting up, while rebuilding the stage or simply while everyone walks to their seats. Within these test set-ups the musicians were also playing, while someone walked across the room or other people were talking to each other.

**Figure A10:**
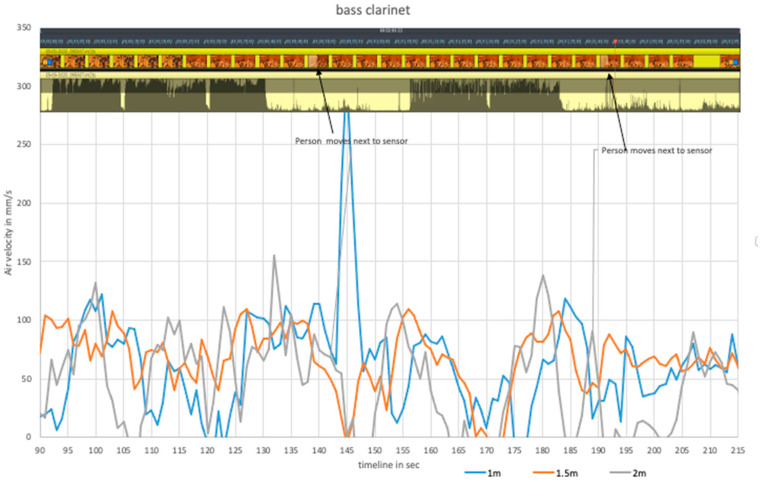
Example measurements of a bass clarinet with the impact of a person passing by the sensor.

From our analysis, we realized that surrounding movements had a serious impact on the measurements, but did not come from the musician while playing. Every movement, before and after playing, led to higher thresholds than measurements while playing. Hence, we differentiated between air velocities coming from the players while playing and the surroundings. It was shown that the air velocities could clearly be separated, since they stayed very close to the instrument’s player (see Appendix A, Figure A10) or came from a disturbing noise, more precisely a disturbing movement. The measurements rose instantly as soon as someone passed the sensors or spoke to the player. Simply raising a hand (making a very small movement) did show air velocity measurements at the closest sensor. This makes the sensor very sensitive to airflow movements, and also explains that there are no significant airflows coming from wind instruments while playing.

The measurements concerning ambient noises were obviously higher than those of measuring individual instrumentalists. They rose up to 0.55 m/s when people around the players were talking, and stayed within a measurement range of 0.18–0.55 m/s when someone walked pass the person playing.

Taking these additional observations into account underlines the article’s argument: that air movements coming from wind instruments while playing are hardly measurable, or if measurable are still much smaller than measurements coming from other surrounding airflows.

## 4. Discussion

This study observed 14 wind instruments played by soloists of the Bamberg Symphony: trumpet, trombone, horn, tuba, alto flute, piccolo, oboe, clarinet, bass clarinet, bassoon, contra bassoon, English horn, saxophone and recorder (the last two instruments by external professional players). Since the measurements with all players were conducted in the concert hall of the Bamberg Symphony, it has a high ecological validity for professional classical music settings.

On the basis of our air velocity measurements, we found that distance regulations of 2 m to the front and 1.5 m to the side are suitable—a finding that is supported by Becher, et al. [2], Kähler and Hain [6], He et al. [8], Mürbe et al. [9] and Parker and Crookston [7]. The values of the analyzed wind instruments mostly did not surpass 0.1 m/s at all distances while playing an excerpt from a music piece, scales or different pitches and volumes, with exceptions for the tuba, reaching a value of 0.13 m/s at 1 m; oboe, 0.15 m/s at 1 m; and contrabassoon, 0.11 m/s at 1 m. These exceptions concern measurements at the 1 m sensor, all of them not surpassing measurements of 0.15 m/s, making the air still a part of the comfortable room air climate [18]. Furthermore, some slight air movements were qualitatively seen at the bells of some wind instruments. These qualitative observations did not reach measurements of more than 0.1 m/s, which is the value of usual room air velocities in hall-like rooms. This observation is further underscored by the consideration of ambient motions because it points out that ambient motions resulted in higher measurements than airflows triggered by the instrument.

Only one study, conducted at the LMU [5], suggests a farther distance regulation of 3 m to the front and 2 m to the side for alto flute players, since the investigators observed farther respiratory air clouds specifically for this instrument. On the basis of our observations as well as the measurements, air-jet woodwinds produce strong side-air movements, which did, in our case, stay within 1 m of the player. Therefore, we cannot confirm the suggestion of 3 m to the front and 2 m to the side, but agree with the fact that side-air movements have to be seriously considered.

Due to their production of side-air movements, the air-jet woodwinds alto flute and piccolo were unique among our findings. They reached high measurements of 0.13 m/s (piccolo) and 0.15 m/s (alto flute) to the side at a distance of 0.5 m. This observation highlights the importance of the structure and the mouthpiece of wind instruments and is relevant as to which wind instrument is taken into account. Becher et al. [2] supported this finding by observing a maximal value of air dispersion at 1.12 m from the mouth of the piccolo player into the room. The measurement from the bell of the piccolo though was around 0.2 m. The high number of 1.12 m came from the side-air, which was released due to way the instrument is overblown. This finding also corresponds with the finding of Becher et al. [2] on the importance of individual blowing techniques for air movements. We thus align ourselves with the assumption of Becher et al. [2] and He et al. [8] that the structure of an instrument as well as the way a mouthpiece is blown have significant influences on the air velocity generated while playing, the distance it reaches and subsequently the generation of aerosols.

Hence, our findings do point out that there is a difference between playing the instrument and warming up the instrument, since warming up produced higher airflow measurements than playing. As with the study of Kähler and Hain [6], we observed that pitch or volume do not have significant impacts on the velocity of air movements. Thereafter, we found out that using only a mouthpiece for warm-up-playing produces strong and fast airflows. Measurements of up to 0.5 m/s were acquired for warming up, in relation to 0.13 m/s for playing (at the 1 m sensor), confirming the suggested visual observations. These warm-ups usually take place in single rooms, before the concert, but they are often conducted during a concert or rehearsal as well. Regarding the high air velocities produced, we strongly suggest not blowing through a mouthpiece (without the instrument) when other musicians are around.

Comparing brass and woodwinds, it was qualitatively seen as well as quantitatively measured that professional brass players do not produce air movements at the mouthpieces, but at the bells of the instruments. Unlike Kähler and Hain [6], we did not observe a severe difference between the production of respiratory air movements between woodwinds and brass. The difference we observed lies more within the structure of the instrument and its mouthpiece, as mentioned above. He et al. [8] also pointed out the difference between brass and woodwinds, which we can concur with but not support completely, since air velocity measurements did not diversify significantly.

As for woodwinds, a difference between so-called air-jet woodwinds and reed woodwinds was not only qualitatively observed, but also quantitatively measured.

The difference between reed and double reed woodwinds was not significant according to our measurements, and was therefore not taken into account. However, it has to be mentioned that reed woodwinds produce little air movements at their tone holes. These air movements were seen in the qualitative observations, but did not reach significant values that would surpass usual room air velocities of 0.1 m/s.

As for the measurements of the oboe, which were comparably high (0.15 m/s at 1 m and 0.12 m/s at 1.5 m), we assumed that surrounding air velocities led to these values. Otherwise, the values are not plausible, considering the way the instrument and the mouthpiece are played, and also regarding our qualitative observation. It can be expected that the measurements should be similar to those of other double reed instruments, such as the bassoon or contrabassoon, which reached 0.1 m/s at 1 m (for the contrabassoon) and stayed below 0.1 m/s beyond 1.5 m (for both double reeds).

Aside from our findings, some limitations of our study have to be taken into account.

First, the study was conducted with highly professional classical wind instrumentalists, and the results can therefore not automatically be applied to other musical genres and settings or amateur musicians. Since the study focused on the dispersion of airflows of individual wind instrument playing, aspects of group playing were not considered. In this sense, it would also be necessary to investigate other parameters influencing air dispersion, such as temperature and humidity caused by the presence of more players and by equipment, e.g., stage lights.

Second, the test situation was rather specific. We took into account different test settings: playing while setting up the stage (concerning noises and movements of the surroundings) and playing with nothing else happening in the area. Furthermore, the difference between playing and warming-up was considered. Therefore, the findings are very representative for orchestras and a high level of playing, with restricted transferability for the amateur music sector. Since the players were a part of the analysis and knew their instruments very well, relevant airflow outputs were identified for each instrument. This seems to be one of the strengths of our study.

Further limitations concern the fact that the measurements were conducted with one person per instrument only, while being sensitive to individual differences of blowing or lung volume, etc. Additionally, they were only performed once for every instrument, whereas more repetitions of the same sequence played would have given more information on reproducibility of the test setting.

Another limitation for the measurement is the fact that the air velocity measurements are very sensitive to surrounding movements, with the waving of a hand already influencing the measurements at the sensors.

## 5. Conclusions

The test results have pointed out that most wind instruments do not have any visual or measurable influence on the movement of compartment air. While playing the alto flute, light flow movements were visible close to the musician’s body. Regarding all wind instruments, beyond a distance of 1.5 m toward the front, no airflows could be measured and therefore no difference compared to usual airflows of hall-like rooms (movie theatres, theatres, auditoriums, opera, etc.) could be found.

Since no respiratory air movements—of any wind instrument analyzed—were measured at the 2 m sensor, we find distance regulations of 2 m to the front of wind instrument players suitable.

We also want to address the point that to better understand the stage situation for larger ensembles especially, it would be important to conduct further studies on air-conditioning of stages, regarding their special climates, and to also observe the impact of many musicians playing together, to find out if and how they influence air velocities.

In order to maintain responsible risk management, we find it crucial that besides distance regulations and line-up (e.g., large ensembles), constant fresh air conditioning and appropriate social behavior should be considered. Starting in April 2020, the Freiburg Institute of Musicians’ Medicine constantly updated an official paper on “risk assessment of a coronavirus infection in the field of music” (updated: 19 May 2020, 1 July 2020, 17 July 2020, 14 December 2020), stating the findings of our various studies publicly [20] and establishing a permanent consultation relationship on questions concerning the relationship between the coronavirus and music making.

## Figures and Tables

**Table 1:** Maximal measurements of test instruments, while playing an excerpt from a music piece.

Instruments	1 m	1.5 m	2 m
brass			
trumpet	˂0.1 m/s	˂0.1 m/s	˂0.1 m/s
trombone	˂0.1 m/s	˂0.1 m/s	˂0.1 m/s
horn	˂0.1 m/s	˂0.1 m/s	˂0.1 m/s
tuba	0.13 m/s	˂0.1 m/s	˂0.1 m/s
woodwinds			
(1) air-jet			
alto flute	˂0.1 m/s	˂0.1 m/s	˂0.1 m/s
piccolo	˂0.1 m/s	˂0.1 m/s	˂0.1 m/s
(2) single reed			
clarinet	˂0.1 m/s	˂0.1 m/s	˂0.1 m/s
bass clarinet	˂0.1 m/s	˂0.1 m/s	˂0.1 m/s
saxophone	˂0.1 m/s	˂0.1 m/s	˂0.1 m/s
(3) double reed			
oboe	0.15 m/s	0.12 m/s	˂0.1 m/s
bassoon	˂0.1 m/s	˂0.1 m/s	˂0.1 m/s
contrabassoon	0.1 m/s	˂0.1 m/s	˂0.1 m/s
English horn	˂0.1 m/s	˂0.1 m/s	˂0.1 m/s

## Data Availability

The data presented in this study are available on request from the corresponding author. The data are not publicly available due to respecting the privacy of persons involved.
